# Veteran-centered barriers to VA mental healthcare services use

**DOI:** 10.1186/s12913-018-3346-9

**Published:** 2018-07-31

**Authors:** Ann M. Cheney, Christopher J. Koenig, Christopher J. Miller, Kara Zamora, Patricia Wright, Regina Stanley, John Fortney, James F. Burgess, Jeffrey M. Pyne

**Affiliations:** 1 Department of Social Medicine, Population, and Public Health, Center for Healthy Communities, School of Medicine, University of California, Riverside, 900 University Avenue, Riverside, CA 92521 USA; 2 Department of Communication Studies, San Francisco State University, 1600 Holloway Avenue, San Francisco, CA 94132 USA; 3 The Center for Healthcare Organization and Implementation Research, VA Boston Healthcare System, 150 S. Huntington Avenue, Boston, MA 02130 USA; 4 Department of Psychiatry, Harvard Medical School, 2 West, Room 305, 401 Park Drive, Boston, MA 02215 USA; 5 San Francisco Veterans Affairs Health Care System, 4150 Clement Street, San Francisco, CA 94121 USA; 6 School of Nursing, University of Arkansas for Medical Sciences, 220 Campus Dr, Little Rock, AR 72205 USA; 7 HSR&D Center of Innovation, Central Arkansas VA Health Care System, 2200 Ft. Roots Drive Bldg. 58 152/NLR, North Little Rock, AR 72114 USA; 8 Health Services Research and Development, Center of Innovation for Veteran-Centered and Value-Driven Care, VA Puget Sound Health Care System, 1660 S. Columbian Way S-152, Seattle, WA 98108 USA; 9 Department of Psychiatry and Behavioral Sciences, University of Washington, 1959 NE Pacific St, Seattle, WA 98195-6560 USA; 10 Department of Psychiatry and Behavioral Sciences, University of Arkansas for Medical Sciences, 4301 W. Markham St, Little Rock, AR 72205 USA

**Keywords:** Cultural domain analysis, Health care services use, Mental health, Patient-centered care, Qualitative research, Veterans

## Abstract

**Background:**

Some veterans face multiple barriers to VA mental healthcare service use. However, there is limited understanding of how veterans’ experiences and meaning systems shape their perceptions of barriers to VA mental health service use. In 2015, a participatory, mixed-methods project was initiated to elicit veteran-centered barriers to using mental healthcare services among a diverse sample of US rural and urban veterans. We sought to identify veteran-centric barriers to mental healthcare to increase initial engagement and continuation with VA mental healthcare services.

**Methods:**

Cultural Domain Analysis, incorporated in a mixed methods approach, generated a cognitive map of veterans’ barriers to care. The method involved: 1) free lists of barriers categorized through participant pile sorting; 2) multi-dimensional scaling and cluster analysis for item clusters in spatial dimensions; and 3) participant review, explanation, and interpretation for dimensions of the cultural domain. Item relations were synthesized within and across domain dimensions to contextualize mental health help-seeking behavior.

**Results:**

Participants determined five dimensions of barriers to VA mental healthcare services: concern about what others think; financial, personal, and physical obstacles; confidence in the VA healthcare system; navigating VA benefits and healthcare services; and privacy, security, and abuse of services.

**Conclusions:**

These findings demonstrate the value of participatory methods in eliciting meaningful cultural insight into barriers of mental health utilization informed by military veteran culture. They also reinforce the importance of collaborations between the VA and Department of Defense to address the role of military institutional norms and stigmatizing attitudes in veterans’ mental health-seeking behaviors.

**Electronic supplementary material:**

The online version of this article (10.1186/s12913-018-3346-9) contains supplementary material, which is available to authorized users.

## Background

Some veterans using healthcare services at the Department of Veterans Affairs (VA), face multiple potential barriers to VA healthcare services, particularly mental healthcare [1, 2]. Previous research has examined the barriers faced by veterans obtaining needed VA mental healthcare services [3–9]. Limited attention has been paid to the influence of veterans’ experiences (e.g., prior military service, day-to-day struggles) and meaning systems on their perception of barriers to VA mental healthcare services.

Patients, physicians, and researchers who have different life experiences and backgrounds may use different paradigms and language—that is, different cultural frameworks when thinking about healthcare or engaging with healthcare systems [10–12]. For example, veterans’ military history greatly influence their understanding of mental health and the language used to describe those experiences [13], while physicians’ and researchers’ biomedical and scientific research training shapes their understanding of health and use of language to describe patient experiences [14]. The VA has made important strides toward veteran-centric care that places the voice of veterans and their unique needs at the center of healthcare. Recently, the VA has implemented and funded a number of initiatives (e.g., Center for Compassionate Innovation, MyVA, Whole Health Initiative), as well as research, trainings, and quality improvement projects to facilitate this culture shift [15–17].

In this era of greater emphasis on patient-centered care approaches, where researchers are encouraged to meaningfully collaborate with populations they study [10], one critical care need is to understand the barriers to care grounded in veterans’ frameworks for thinking about health. Veterans with mental health problems tend to perceive more barriers to mental health treatment than veterans without similar problems [18–21]. By understanding veteran-centric barriers to mental healthcare, we can better understand how to increase initiation and engagement with VA mental healthcare services, as well as adherence to mental health treatments.

In this paper, we report on the use of cultural domain analysis (CDA), a method common in ethnographic research [22], to elicit veteran-centered concepts of barriers to VA mental healthcare services use. We selected this approach over quantitative methods such as survey questions because it allows veterans to define barriers to care that they themselves experience, and to talk about how their experiences influence their mental health service use. Using a series of elicitation and interpretation interviews, we elicited veteran-centered barriers to care and then asked veterans to explain the meaning of these barriers in their own VA healthcare experiences. CDA methods elicit an “emic” perspective (the perspective of the participant), rather than an “etic” perspective (the perspective of the researcher) [23, 24]. This analysis is the initial phase of a sequential mixed-methods study designed to develop and validate a perceived access measurement tool. The first phase of the larger study involved qualitative semi-structured interviews with veterans, of whom a subsample participated in the CDA and domain interpretation interviews. This paper reports on these findings.

## Methods

We used standard CDA procedures to elicit veteran-centered barriers to mental healthcare and to create a cognitive map [25, 26]. We followed up with qualitative, semi-structured interviews that were conducted during one-hour interview sessions to understand the meaning of these barriers in veterans’ healthcare experiences. For all interviews, participants completed a brief quantitative sociodemographic survey and reported on their current mental health symptoms severity for depression, post-traumatic stress disorder (PTSD), and alcohol use. Depression severity was measured using the Patient Health Questionnaire nine-item depression module (PHQ-9) from the PRIME-MD [27–29]; PTSD symptom severity was measured using the PTSD Checklist Civilian version (PCL-C) [30]. At-risk drinking was assessed using the AUDIT-C [31]. All participants provided written informed consent and were remunerated for their study participation. Prior to the start of the research, the VA’s Central Institutional Review Board approved all study procedures.

### Setting

This study was conducted at ten sites in three geographically distinct VA administrations areas called Veterans Integrated Service Networks (VISN*s*): VISN 1 included one urban and two rural CBOCs in Maine serving primarily white veterans; VISN 16 included one urban health clinic and two rural CBOCs in Arkansas serving a largely white and African American veteran population; and VISN 21 included one urban and three rural CBOCs in Northern California serving a racially and ethnically diverse group of veterans. Table 1 shows the rural, urban, and ethnic/racial breakdown across sites.

**Table 1:** Free list items within their dimensions of the cultural domain

Dimension 1	Dimension 2	Dimension 3	Dimension 4	Dimension 5
*Worry and concern about what others think*	*Financial, personal, and physical obstacles*	*Confidence in the VA healthcare system*	*Navigating VA benefits and healthcare services*	*Privacy, security, and abuse of services*
Racism (31)^a^	Homelessness (14)	No follow-up from doctors or counselors (28)	Lack of knowledge and understanding (43)	Fearing of losing security clearance (45)
Stigma (32)	Health, injury, sickness (26)	Problems with scheduling appointments (13)	Concerns about getting mental healthcare (4)	Fear of using video teleconferencing (example: V-tel) (9)
Judgment (30)	Having other priorities (example: life getting in the way) (12)	Too few providers and staff (15)	Paperwork is daunting (example: VA referral forms are hard to fill out) (33)	Losing rights fought for (39)
Perception of mental healthcare (20)	Not having steady employment (36)	Lack of available treatment (16)	Outreach about VA healthcare (3)	Veterans abusing system (29)
Civilians not understanding what veterans have gone through (11)	Legal issues (1)	No follow-up from VA (40)	Problems with getting service connected disability (23)	
“Suck it up” mentality (6)	Finances (example: not having enough money) (37)	Appointments are hard to get (10)	Mental health not asked about in primary care (5)	
Friends, family, doctor thinking you’re “crazy” (38)	Childcare (44)	Clinics respond slowly to Veterans in crisis (25)	Knowledge of where to go and who to contact for mental health services (18)	
Trust (41)	Cost of travel (46)	Providers changing jobs or leaving (35)	Knowledge of what kinds of mental health treatments are available (27)	
Worry about what others think (24)	Travel distance (19)	Waiting for appointments (34)		
Weakness (example: feeling like a failure or weak) (42)	Transportation problems (7)			
Anxiety (22)				
Alcohol and drug use (8)				

^a^Numbers beside each phrase represent items consolidated from the free list exercise and presented to veterans during the pile-sort activity. These numbers were used for the Multi-Dimensional Scaling and Cluster Analysis

### Recruitment

We recruited veterans who used VA healthcare and had behavioral health concerns, but who did not necessarily use specialty mental health services. Eligible participants included veterans between 18 to 70 years old who screened positive for at least one behavioral health problem, including depression, PTSD, or at-risk alcohol use during a routine VA primary care appointment within the past year. We used opt-out letters, a proven strategy for gaining veterans’ participation in health services research [32–34], to recruit a diverse sample of veterans across the three states.

As described in detail elsewhere [35, 36], we sent opt-out letters to anywhere from 30 to 60 eligible veterans within each of the three VISNs repeating this procedure in three successive waves. Opt-out letters were sent to 585 veterans across all three sites. Eligible veterans received a letter describing the study and the opt-out process that involved returning a self-addressed, stamped, “do not call” form or calling a 1–800 number to opt-out. A total of 89 veterans opted out of the study. Within two weeks of receiving the letter, study staff called the remaining 496 veterans who did not opt-out. Veterans who had access to a telephone and had experienced mental health problems related to PTSD, depression, or alcohol use were invited to participate in the study. An additional eight veterans had already been recruited into the study during a pilot phase to assess the study design. We called to confirm eligibility and discuss study participation; 258 veterans were reached and 72 participated in the study. The final study sample included 80 veterans.

Based on preliminary analyses and review of our recruitment log, we then purposively identified certain subpopulations of veterans (e.g., rural veterans, Iraq and Afghanistan veterans, women veterans) to recruit, ultimately diversifying our sample. In wave 1, we recruited veterans of differing service eras obtaining representation of older and younger veterans (e.g., Vietnam veterans, Iraq and Afghanistan veterans); in waves 2 and 3 we recruited veterans based on race and ethnicity. For all waves, we recruited veterans from both rural and urban contexts and over-recruited women veterans, an underrepresented veteran subpopulation.

Of the 80 veterans in the sample, 66 veterans participated in the cultural domain analysis and interpretation interview. As described below, this method requires a relatively small sample size. Therefore, we recruited participants into the CDA phase until agreement was reached (*n* = 52) and into the domain interpretation task until saturation was reached (*n* = 14).

### Cultural domain analysis and interpretation interview

Cultural Domain Analysis (CDA) examines how members of a group who share a culture characterize aspects of that culture through a cognitive domain, referred to as a cultural domain in CDA, defined by a set of words, phrases, or concepts that collectively symbolize a single idea [37]. The CDA method elicits participants’ words and brief phrases that constitute a domain, analyzes its structure, and assigns those structures into one or more dimension. CDA is a multi-step, iterative mixed-methods approach that intersperses data collection with analysis. Analysis of each step systematically informs the next step to ensure a comprehensive interpretation. Sample size determination for the CDA steps is based on inter-correlations among participants about domain items and requires very small sample size; and high domain agreement can be reached with as few as 15 participants per step [38, 39]. We added a final phase of qualitative interviews to validate domain dimensions and elicit veterans’ understandings of the meanings of identified barriers in veterans’ healthcare experiences [40]. We describe each step of the CDA process below.

*Free list exercise.* We started with a free list exercise, an unstructured, open-ended task used to elicit the words and brief phrases that inductively characterize a domain. During data collection, each participant was asked to “list all the things that you think can make it more difficult for veterans to get help for stress-related and emotional health problems (see Additional file 1 for a copy of the guide). Participants listed as many items as they could in response to the verbal prompt. To elicit additional items, interviewers used common free list probing techniques, including non-specific prompts, such as “What other kinds of things make it more difficult to get help?”; reading back free lists to check for accuracy, followed by non-specific prompts; and semantic cues, such as “What about [transportation], can you think of anything else that is similar to this item?” [41]. A total of 23 veterans across the three VISNs participated in the free list task.

Participants’ responses included a total of 238 unique single-word items and brief phrases. The qualitative team reviewed the items, eliminated duplicates, and combined similar items to reduce the list; calculated the frequency of items; and selected items with a frequency ≥ 5 and items with lower frequency but high importance based on qualitative analyses of interview data [42]. Table 1 shows the master list of 46 unique items generated by this data reduction process and used in the subsequent pile sort activity.

#### Pile sort activity

After compiling the free lists, we asked a new set of participants to conduct a pile sort activity in which participants organize or sort a finite number of discrete words and phrases into categories according to whatever criteria they see fit. The 46 items generated in the free list exercise were placed on index cards and assigned a unique number. Interviewers asked participants to “place all the items you think are similar into piles,” using as many or as few piles as necessary. Finally, participants were asked to review and label each pile with a descriptive title. A total of 29 veterans across the three states participated in the pile sort activity.

**Fig. 1:**
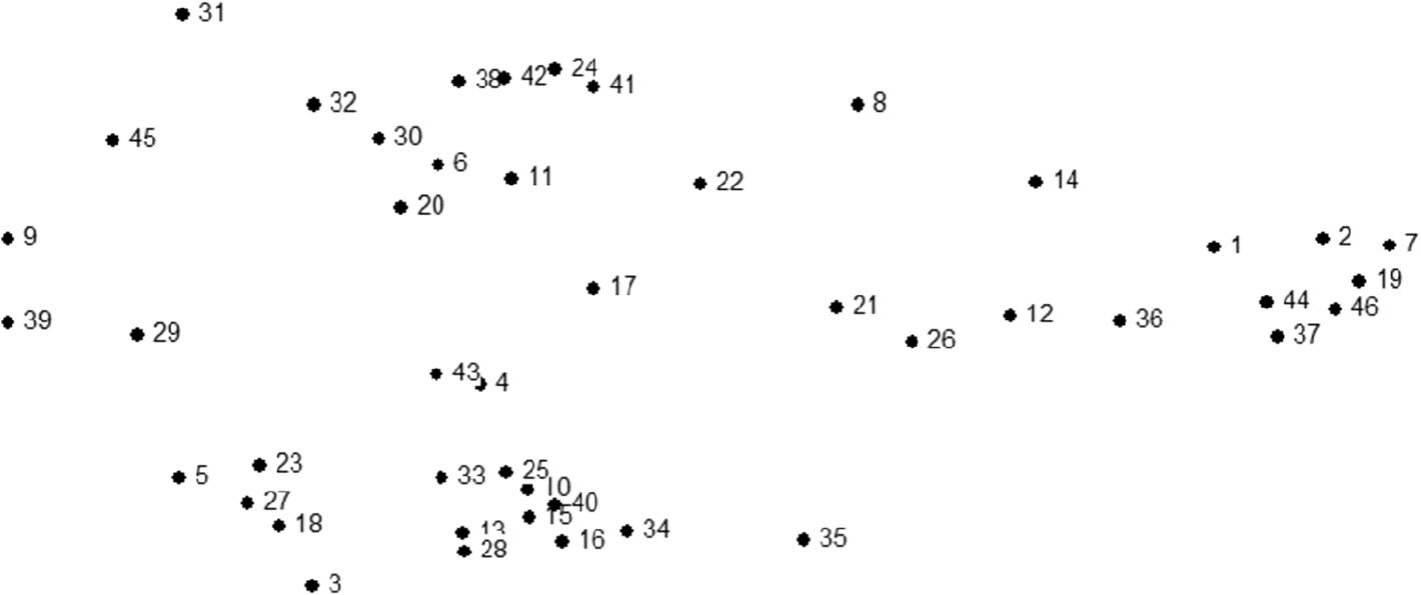
Multidimensional scaling of free list items

**Fig. 2:**
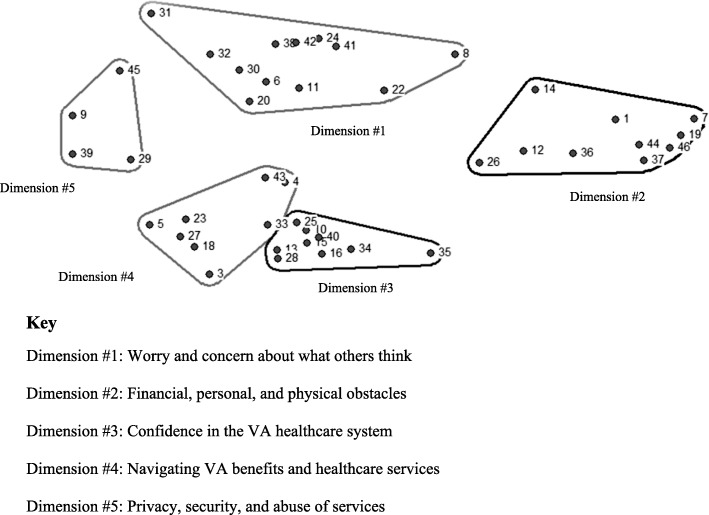
Cultural dimensions of access barriers

Pile sort data were entered into a database keyed on index card label and unique number and were imported into Anthropac, an analytic program designed to collect and analyze cultural domain data by identifying underlying relations between items and meaningful group clustering [43]. Pile sort data were analyzed using the Anthropac multidimensional scaling (MDS) tool to identify each statement as a separate point on a two-dimensional map, which provides a visual representation of participants’ sorting data as illustrated in Fig. 1 [44]. MDS graphs prioritize meaning through spatial proximity (i.e., items appearing closer together are more conceptually similar; items appearing farther apart are more conceptually distinct). MDS calculates a stress value, a diagnostic statistic measuring the overall fit of the map (i.e., goodness-of-fit indicator). Lower stress values more accurately reflect congruence between the raw data and the computed distance matrix. For example, 0.0represents perfect congruence between the raw data and the distance matrix whereas ˃0.2 represents poorer congruence and goodness of fit [45]. The average estimated stress value for similar studies using MDS to analyze pile sort data is .280 [46]. We obtained a stress value of .181, representing a good fit of the map to the data. Finally, we conducted a cluster analysis of the MDS to identify meaningful groupings of items across participants as shown in Fig. 2 [47].

#### Domain interpretation task

Finally, after generating MDS and cluster analyses, we asked a third set of participants to examine the results as a means to validate the domain and its dimensions and understand the meaning of the dimensions and constituent items in veterans’ VA healthcare experiences. We asked participants to explain why items clustered together and their relationship to veterans’ healthcare experiences. We then asked veterans to label each pile with a unique descriptive title they created to characterize the pile. This resulted in different descriptive titles from the pile sort exercise. A total of 14 veterans across the three VISNs participated in this phase.

Participants’ responses during the interpretation task were audio-recorded, professionally transcribed, and imported into qualitative data management software [48]. Following the analytic process described by Ryan and Bernard [49], the first author read the transcribed text line-by-line to identity themes, writing memos throughout. A coding schema was developed with code definitions, and thematic codes were applied to narrative text [50]. Axial coding was used to make comparisons across transcripts and to understand the relations among themes within and across transcripts [50]. The qualitative team reviewed the resulting analysis, and the full qualitative team contributed to and decided on final cluster names through an iterative process.

## Results

Sixty-six unique veterans between the ages of 20 to 70 (average 44.0) years participated in one of the three CDA phases (See Table 1). Most participants were male (*n* = 50), white (*n* = 40), and had prior specialty mental health visits (n = 40); 24 screened positive for PTSD, 28 for depression, and 40 for at-risk drinking. All had experience using the VA healthcare system; consequently, their understanding of barriers to care was grounded in their experiences.

The cultural domain is presented in Fig. 2. Numbers denote the items in the domain dimensions corresponding to the item numbers in Table 2. In MDS, items closer together are more closely related to one another; and in hierarchical cluster analysis, clusters of items are placed together to represent dimensions of the larger cultural domain.

**Table 2:** Participant characteristics

*Variable*		*Veteran Integrated Service Network (VISN)*							
	Yes/No	VISN 1 Maine (*N* = 24)		VISN 16 Arkansas (*N* = 21)		VISN 21 California (*N* = 21)		Total (*N* = 66)	
CDA Phases		N		N		N		N	
Free List		7		7		9		23	
Pile sort		12		10		7		29	
Interpretation task		5		4		5		14	
Prior mental health visit	Yes	14		14		12		40	
	No	10		7		9		26	
Gender									
Male		17		16		17		50	
Female		7		5		4		16	
Ethnicity									
Hispanic/Latino	Yes	1		4		4		9	
	No/missing	23		17		17		57	
Race									
American Indian	Yes	0		2		3		5	
Asian	Yes	0		0		3		3	
Hawaiian/Pacific Islander	Yes	0		0		1		1	
Black	Yes	1		11		0		12	
White/not-Hispanic	Yes	20		7		15		42	
White/Hispanic	YES	0		3		4		7	
Multi-racial	Yes	1		0		1		2	
Other	Yes	1		0		1		2	
Age	Mean	47.4		39.5		45.2		44	
Employment	Student	2		1		2		5	
	Homemaker	1		0		0		1	
	Disabled	10		6		5		21	
	Full-time	6		11		8		25	
	Part-time	1		2		2		5	
	Sick leave	0		0		1		1	
	Other	0		0		1		1	
	Retired	3		0		0		3	
	Unemployed	1		1		1		3	
	On SSI/SSDI	0		0		1		1	
Service disability	Yes	8		5		3		16	
	No	1		0		0		1	
	N/A	14		15		15		44	
	Don’t know	1		1		3		5	
Income	0–50,000	16		15		15		46	
	50,001+	7		6		6		19	
	Refused	1		0		0		1	
Alcohol	Yes	15		9		16		40	
PTSD	Yes	7		11		6		24	
Depression	Yes	8		12		8		28	

The CDA procedures resulted in five dimensions of barriers to care: worry and concerns about what others think; personal, financial, and physical obstacles; confidence in the VA healthcare system; *n*avigating VA benefits and healthcare services; and, privacy, security, and abuse of services. (These labels represent the most common words and phrases veterans used to label each dimension.) Figure 2 provides a visual representation of domain dimensions and Table 3 provides narrative text to further explicate the meaning of domain items to veterans’ healthcare experiences.

**Table 3:** Themes in veterans’ interpretations of the five dimensions of barriers to care continuum

Theme	Speaker	Quote
Dimension 1: Worry and concern about what others think		
*Stigma*		
	Urban veteran in their 40s, positive screen for alcohol misuse or abuse, with prior VA mental health visits	“I think judgment and stigma is all perception about mental health. That’s all not knowing what you are talking about. People thinking you are crazy, not understanding and then use of drug and alcohol just to numb that.”
	Rural veteran in their 60s, positive screen for depression, with prior VA mental health visits	“You’re not able to get help because you’re afraid of the stigma put on you by the outside world.. .. They all make sense to me. I’ve gone through many of these because of my alcohol and drug use. If I tell ‘em, “No,” they [healthcare professionals] keep pressuring me and pressuring me. And I get tired. So yeah, I smoke a joint every once in a while. I only smoke one joint. It seems to satisfy them.”
	Rural veteran in their 30s, positive screen for alcohol misuse or abuse and depression, with prior VA mental health visits	“They [military leadership] tell you from the get go that once you go over there [mental healthcare services], it follows you.. . So then you think you’re gonna carry the stigma of being a mental health patients just ‘cause you went by there.”
	Urban veteran in their 20s, positive screen for PTSD, with prior VA mental health visits	“It’s scary to go for mental healthcare because people automatically think that you are not stable enough to handle tasks in the military. And then if you say that you are seeking mental healthcare, they automatically write you off as not being capable. They don’t think that you are worthy or that they automatically just start discrediting you because you are seeking help. And in the military, they don’t think you should be able –they almost don’t want you to seek help. Even though they tell you to, they say the right things, but the actions of people who are appointed over us, their actions are a totally different way, and they back themselves up with paperwork and regulations, and they keep people scared.”
*Weakness*		
	Rural veteran in their 30s, positive screen for alcohol misuse or abuse and depression, with prior VA mental health visits	“I guess the perception of healthcare, but I was getting healthcare while I was in the military. I would rather talk to a civilian than an actual military person, because I thought the military person in their head was thinking, ‘Suck it up, get back to what to you got to do.”
	Rural veteran in their 60s, positive screen for alcohol misuse or abuse, with no prior VA mental health visits	“When I look at this [Group 1] I guess the title, ‘What names and label would you give this group of items’ [says to self], is something like… ‘Why I wouldn’t seek mental health care.’ I’ve always been worried, prior to-- what I did was with my employers, if they found out it was going to affect my job. I was afraid that I would either be passed over or thought less of. I worry about what others think, that’s me. I feel it is a weakness. It gives me a certain amount of anxiety.”
	Rural veteran in their 50s, positive screen for alcohol misuse or abuse, with no prior VA mental health visits	“I don’t know why [but], suck it up mentality sticks out in my [mind],- because it’s,- so many times I’ve been told to ‘Suck it up,’ and just drag on. Don’t really care, we can’t address your issue. We can’t deal with you. You need to just – Let’s just wait until everything’s over with.. . Hopefully it’s all over with and you don’t need to come talk about it anymore.”
*Trust*		
	Urban veteran in their 40s, positive screen for PTSD and depression, with prior VA mental health visits	Interviewer: Are there other things you find yourself worrying about in accessing services that aren’t listed there? “I would say, ah, worrying about being put in a mental hospital. *Interviewer: Worrying about hospitalization?* “Yes.”
	Rural veteran in their 60s, positive screen for depression, with prior VA mental health visits	“So, learned to adjust to it because nobody believed me. So there again, trust. Some of us [veterans] we lie and some of us we lie very well, because I’ve seen guys here [VA] and I’ve seen ‘em at home, on the street, and it’s a different thing. I said [to other veterans], ‘Well, don’t ya remember [when you? 0:12:41].’ They say, ‘Aw that’s a different thing.’ I said, You lying, man.’ ‘No, I ain’t lying,’ [responds other veteran]. And I leave it alone because I don’t want anybody to ask me any questions, you know?”
Dimension 2: Financial, personal, and physical obstacles		
*Responsibilities*		
	Rural veteran in their 50s, positive screen for alcohol misuse or abuse, with no prior VA mental health visits	“To me it does because homelessness, health and injury, sickness. You could put that, this is kind of like why you’re there, not having steady employment, wow legal issues, wow that could go right up there. Having other priorities in life, I don’t know. Finances, childcare, cost of travel, travel distance equals cost of travel, transportation problems could equal distance, so I mean travel, all those thing could go together. All those are monetarily, personal, but these are all to me, personal issues.”
	Urban veteran in their 20s, positive screen for PTSD, with prior VA mental health visits	“Homelessness, all of it. It’s everything that kinda are like big pictures to people, to me even. Cost of travel and childcare and finances. It’s just an overwhelming sense of, ‘I have so much responsibility, I don’t have help.’”
	Urban veteran in their 40s, positive screen for PTSD and depression, with prior VA mental health visits	“Kinda thinkin’ the ‘health, injury, and sickness’ [doesn’t fit], but in a way it does kind of tie in with finances ‘cause if you get hurt then you’re out of a job.”
*Physical obstacles*		
	Urban veteran in their 30s, positive screen for depression, with prior VA mental health visits	“Even transportation problems if you don’t have transportation and the VA clinic is far – you’re not gonna get there.”
	Urban veteran in their 40s, positive screen for alcohol misuse or abuse, with prior VA mental health visits	“I would say these are physical barriers [cost of travel, travel distance, transportation problems] to coming, getting help.. .. This might be a problem [finances] because you don’t know, you don’t have any steady income, you don’t know how much it’s gonna cost, I mean even if you’re a veteran.. .. I would say these are physical barriers to coming, getting help.”
Dimension 3: *Confidence in the VA healthcare system*		
*Appointments*		
	Rural veteran in their 60s, positive screen for depression, with prior VA mental health visits	“When you say a problem with the system – you don’t know where the system starts and ends at.”
	Urban veteran in their 40s, positive screen for alcohol misuse or abuse, with prior VA mental health visits	“That’s the problem with the VA, sometimes you have to wait three weeks, sometimes they don’t get you in right.. .. My boss, he’s always complaining about the VA, you know, how they misdiagnose and they just gave him the runaround, “Go to this office,” and then this guy says, ‘Go to that office.’”
*Staffing*		
	Urban veteran in their 40s, positive screen for alcohol misuse or abuse, with prior VA mental health visits	“The VA just seems like they’re always short-staffed. I know because my unit is always short-staffed.”
	Rural veteran in their 60s, positive screen for alcohol misuse or abuse, with no prior VA mental health visits	“My experiences—providers changing jobs, that’s been a problem for me. I’ve had a number of doctors going back and forth that have moved.”
*Follow up*		
	Rural veteran in their 60s, positive screen for depression, with prior VA mental health visits	“I follow up, but sometimes it would be such a long time. And I will call and check and if I’m coming up here for something else I will. If I’m able to walk in – before I got these I used to have to drag myself along the rail and it has a lot of germs.”
	Rural veteran in their 30s, positive screen for alcohol misuse or abuse, with no prior VA mental health visits	“These are all just stuff that, I hear this from people all the time. I just had a buddy of mine - his dad was a Korean veteran—he was havin’ all kinds of intestinal problems but didn’t know how to use any of the digital parts of the VA. Didn’t know how to get help and have somebody advocate for him or anything like that. And this guy was completely stressed out ‘cause his dad was gettin’ treated like shit in the VA because he didn’t know how [to navigate the system], and I gave him one phone number for DAV, and the advocate called him up and was like, ‘Hey, here ‘ya go.” Boom, boom, boom, boom. Within a week the guys care was completely turned around. Just because you gotta know the right channels. If you aren’t doing the rights things then you’re gonna get screwed with the VA.”
	Urban veteran in their 40s, positive screen for alcohol misuse or abuse, with prior VA mental health visits	“People are going to slip. You gotta catch them before they get out.”
*Appearance of priority care*		
	Urban veteran in their 20s, positive screen for PTSD, with prior VA mental health visits	“People who retire are often discredited when they go to seek help. People who are in the military are seen a lot quicker and faster, whereas veterans have to wait or they are put to the side--they are not a high priority.”
	Rural veteran in their 60s, positive screen for depression, with prior VA mental health visits	“If they don’t follow up—. .. And, when they [VA providers] tell me that [providing care for younger veterans] I just, back up. ‘Cause I’m thinking they’re taking care of someone they [the government] can use. They can’t use me, I’m too old.”
Dimension 4: *Navigating VA benefits and healthcare services*		
*Benefits*		
	Rural veteran in their 60s, positive screen for depression, with prior VA mental health visits	“This is the most important one, problems with getting service connected disability.. .. But the major one is learning how to get in contact [about benefits]. And when I applied this person [patient advocate] was workin’ with another DAV [Disabled American Veterans].. .. And, I went home and when I come back he [patient advocate] said, ‘You get started?’ I said, ‘No, I was turned down.’ I didn’t even know why. And so whatever he [patient advocate] did, within about three months I had something started then. But only because he did it. So this is a major problem.”
	Rural veteran in their 30s, positive screen for alcohol misuse or abuse, with no prior VA mental health visits	“I mean I know that I can come here [to VA], but I have no knowledge as to what my benefits are. I know I have benefits, but I just don’t know what those are or how I can use ‘em.”
	Urban veteran in their 40s, positive screen for alcohol misuse or abuse, with prior VA mental health visits	“Yeah, so my experience with the VA was better than most veterans. I was in Korea and they had a VA office there, so I did all my paperwork and I got all my information before I got out, so I was good. But the problem is that a lot of veterans don’t know that these resources are available. And so yeah, these all make sense.. .. And you know, no one wants to get mental health, there’s a stigma. So I’m gonna say, yeah this goes together, like information, like the VA has to be better at distributing information.. . It’s better to hand it out to people before they leave [the military], ‘cause once they leave the system it’s kinda hard to track ‘em down. People just disappear. So I don’t know how you would -- You could do outreach to rural areas, but someone living in Montana somewhere, I don’t know how you would reach out to them.”
*Mental health services*		
	Rural veteran in their 30s, positive screen for alcohol misuse and abuse and depression, with prior VA mental health visits	“‘Cause the VA doesn’t tell you about the kinds of [mental health] treatments that are available.”
	Rural veteran in their 60s, positive screen for alcohol misuse or abuse, with no prior VA mental health visits	“I haven’t had the personal experience about going after the mental health care as I didn’t go to my VA to get my benefits until late in life.. .. I would think that a lot of people would have the lack of knowledge and understanding of how it works.. .. And I guess there probably could be a lot more knowledge about what kinds of mental health treatments are available, but I’ve never asked. So I don’t know what the brochures are and how… But I think it’s if you’re really there, you’re gonna find out, and or, there will [be] some key thing that comes up.. .”
	Urban veteran in their 30s, positive screen for alcohol misuse or abuse, PTSD, and depression, with prior VA mental health visits	“It’s like the lack of transparency over mental health as far as, informing the people that need the services what services are provided.”
	Urban veteran in their 30s, positive screen for depression, with prior VA mental health visits	“Lack of information, I guess? I mean we didn’t even know we were able to come to the VA until we were veterans for like 5 or 6 years. So for 5 or 6 years I was paying on my own for my mental health care ‘cause I had no idea that I could come here and get my medications and everything, so.”
Dimension 5: Privacy, security, and abuse of services		
*Confidential care*		
	Rural veteran in their 30s, positive screen for alcohol misuse and abuse and depression, with prior VA mental health visits	“Fear because of what others say. You heard they didn’t – In the military, that you’ll lose your security clearance if you go to mental health.”
	Rural veteran in their 50s, positive screen for alcohol misuse or abuse, with no prior VA mental health visits	“I am not doing any kind of mental health, whatever, because everything you guys [VA providers] do, you type and you put it in this computer. And they said, ‘No, this is strictly confidential. Nobody will ever know.’ I said, ‘Well, what if the backing system just got hacked.’”
	Rural veteran in their 60s, positive screen for depression, with prior VA mental health visits	“I was telling VA – I mean combat stories which I don’t tell. It’s been too long.. . When they [VA] ask these questions, ‘Why don’t you answer the questions?’ ‘Because I’m afraid.’. .. I might go to jail and be locked up too. That what I’m thinkin’ now. It’s not all over with.” – Vietnam Veteran
	Urban veteran in their 30s, positive screen for depression, with prior VA mental health visits	“Being in active duty there is definitely punishment almost for asking even to receive mental healthcare. So I can see that you would be afraid of losing those things.”
*Abuse of services*		
	Rural veteran in their 60s, positive screen for alcohol misuse or abuse, with no prior VA mental health visits	“And there’s a lot of guys that are working the system here. How they manipulate to try and get PTSD… But, maybe there [are], you know, you hear the stories [about veterans manipulating the system] and all of that. So, I don’t know what level I’m at, or where I’m at with things. All I know is, I have friends that – I call it walking a tight rope between reality and non-reality in what you’re doing.”
	Rural veteran in their 60s, positive screen for depression, with prior VA mental health visits	“Because when they took me off my pain medication, I said, ‘Doc, you always tell us not to stop taking the medication until the doctor tell you. You done took all my medication that I’ve been getting for three or four years.’ But, they can never prove that I abuse it, and now, I found out that – from upstairs that it was being cut. Then I see some guys getting medication, ‘I’m gettin’ high tonight.’. .. I said maybe they have another problem, a different problem than I have. ‘You’re going to save me [from addiction]– but I’m still in pain.”

### Dimension 1: Worry and concern about what others think

Veterans’ discussion of this dimension grouped around stigma, vulnerability, and trust as barriers to VA mental health services use. Veterans expressed concern over stigmatizing labels such as “crazy” and “mental health patients” as well as military attitudes (e.g., “suck it up”) fostering feelings of weakness and failure. Such attitudes contributed to veterans’ anxiety and alcohol and drug use and impeded mental healthcare services use. Veterans also indicated a lack of trust in the clinical encounter (e.g., interactions with non-military healthcare providers with limited understanding of veterans’ military experiences) decreased their motivation to remain in care.

### Dimension 2: Financial, personal, and physical obstacles

Discussions centered on personal life struggles (e.g., poor health, legal and financial issues, and homelessness) constraining on veterans’ daily lives and ability to meet responsibilities—including healthcare services use. Travel distance to VA facilities and lack of transportation or gas money also represented barriers to VA care within this dimension.

### Dimension 3: Confidence in the VA healthcare system

Veterans’ discussions of this dimension indicated system-wide problems with VA processes of care and expressed their lack of confidence (e.g., availability of timely visits, follow-up care). Appointment problems, staffing issues, limited follow-up from VA healthcare providers and staff, and prioritization of some veterans over others impeded access to care. Long wait times between appointments, slow clinic response, and challenges to scheduling appointments (e.g., being passed from clinic to clinic) created barriers to timely and appropriate care. Staff issues, especially lack of available providers and high provider turnover, limited veterans’ access to care and provider continuity over time. Veterans also indicated that limited or no access to specialized care resulted in veterans’ disengagement with care. Furthermore, the appearance that services are prioritized to address the needs of younger veterans limits available services for older veterans and diminishes the importance of older veterans’ health and wellbeing.

### Dimension 4: Navigating VA benefits and healthcare services

Veterans’ discussions highlighted the challenges of navigating VA benefits and healthcare services, which are connected to lack of understanding or misunderstanding of VA benefits and mental healthcare services. Veterans reported being unaware of VA benefits prior to leaving military service, having limited knowledge of the processes required to obtain benefits (e.g., necessary paperwork) once eligible, and struggling to understand the use of their benefits as veterans. This knowledge gap impeded initial enrollment into the VA healthcare system. Once veterans entered the system, they still struggled with a lack of awareness of available mental healthcare services. Veterans stressed the need for both the military and the VA to disseminate benefit material more effectively to service members prior to military exit.

### Dimension 5: Privacy, security, and abuse of services

Veterans’ discussions of this dimension revealed concerns regarding healthcare privacy and misuse of the VA healthcare system by other veterans. Veterans discussed military policy denying security clearances for service members known to seek mental healthcare and this fear influencing veterans’ decisions to seek specialty care within the VA healthcare system. Some also expressed a fear of their confidential information being shared and sensitive information (e.g., actions in combat, mental health diagnoses) disclosed, which could have profound negative consequences on veterans’ lives (e.g., incarceration, loss of security clearance). Both older (Vietnam era) and younger (Iraq and Afghanistan era) veterans’ expressed distrust of the VA healthcare system as a government institution and fear of digital communications (e.g., tele-psychiatry). Last, misuse of VA services by some veterans for disability payments and pain medication, while perceived by veterans as rare, had a negative effect on the VA care experience of other veterans. Specifically, misuse of the system influences how society and some providers view veterans who use VA healthcare for certain treatments (e.g., pain medication and mental health treatment).

## Discussion

This mixed-methods CDA analysis generated veteran-centered perspectives of barriers to VA mental healthcare services use grounded in veterans’ lives and during clinical interactions that add depth, breadth, and complexity to understanding barriers to care. This approach contributes to better understanding of veteran-derived perspectives on barriers to mental healthcare services use. The findings are intended to inform patient-centered care [51] and conceptual models, such as the SOTA Access Model [52], and measurement tools [20].

Our interpretation of veterans’ perceptions of barriers to VA service use demonstrates *that* historical, socio-cultural, and psychological factors inform veterans’ decisions to engage with VA mental healthcare services. Their perceptions highlight how military socialization, command structure influences, and institutional attitudes (e.g., “suck it up” mentality) reinforce attitudes around help-seeking as weakness, which significantly informs veterans’ post-military service healthcare experiences [7, 53]. The findings suggest veterans and active duty service members experience similar system-level and socio-cultural barriers to use of government mental healthcare services. In their study of barriers to engaging service members in the Department of Defense (DoD) mental healthcare services, Tanielian et al. [54] found concerns over military healthcare system capacity and processes of care (e.g., limited providers, problems scheduling appointments) and the military ethos of “toughing it out” greatly influenced active duty services members engagement with military mental healthcare services.

In addition, our findings show how institutional betrayal, or as veterans explained, distrust of the VA as a government institution, influences their VA mental healthcare services use. Desai et al. [5] found negative perceptions of social and governmental institutions created barriers to seeking VA mental healthcare services among Vietnam-era veterans. Similarly, veterans in our study, who represented Vietnam, Persian Gulf, and Iraq and Afghanistan service eras, distrusted providers’ use of computers to document personal and sensitive information, questioned the security of digital communications (e.g., video-conferencing), and feared hacking of electronic medical records.

This study also provides insight into ways veterans perceive misuse or abuse of the VA healthcare system. The VA is well aware of potential abuse of its healthcare services. For instance, the VA Office of Community Care has a webpage with frequently asked questions about fraud, waste, or abuse of VA healthcare services (https://www.va.gov/COMMUNITYCARE/about_us/POI/poi_faq.asp). Veterans’ abuse of VA healthcare was present in our study—in the case of a participant retelling how veterans scheme VA doctors to obtain pain medication only to misuse their prescription. Whereas fraud was present in the case of a participant explaining that some veterans manipulate the system to obtain a PTSD diagnosis. Though veterans described them as rare, these practices can shape veterans’ and providers’ attitudes and impede development of a trusting therapeutic alliance between providers and veterans utilizing VA healthcare services.

Our findings provide an evidence base for DoD and VA health policy. We found that norms and values embedded within the military perpetuate stigmatizing attitudes around mental health treatment seeking and continue to influence veterans’ healthcare utilization decisions post military service, and that veterans need support transitioning from the DoD to VA healthcare system. This finding reinforces the need for ongoing collaborations between the VA and DoD to critically address military institutional norms and attitudes stigmatizing service member’s mental health-seeking behaviors and facilitate care coordination as active-duty service members transition to veterans.

Policy to reduce stigmatizing attitudes about mental health and treatment seeking in the military and healthcare transition processes that link service members to VA care prior to leaving the service would be ideal. The VA/DoD “One Mission- One Policy- One Plan” (a community of practice) facilitating service member and veteran care coordination is a step towards reducing barriers to care for younger veterans [55]. Similar communities of practice, such as VA and community-based organizations serving veterans, for older generations of veterans who are not using VA benefits and healthcare services are needed [56].

Furthermore, our findings regarding veterans’ confidence in the VA healthcare system points to service members and veterans’ mistrust in government healthcare. Both VA and DoD should work together to address veterans’ mistrust of the VA healthcare system, which reflects both current public relation issues and the historical treatment of veterans of different service eras (e.g., Vietnam veterans vs Iraq and Afghanistan veterans) within the VA healthcare system [57, 58]. Furthermore, as the use of electronic medical records, digital communications (e.g., tele-psychiatry, secure doctor-patient messaging), and smartphone applications evolves through the twenty-first century, veterans’ distrust of the VA healthcare system as a government institution may impede use of technology which is important issue to address for some veterans.

Several limitations should be considered when interpreting the findings. Our findings reflect insight from veterans enrolled in VA healthcare services and may not reflect the experiences of veterans who have not used VA benefits and services. While we had much success recruiting participants from both rural and urban settings across the three sites (Arkansas, California, and Maine), we enrolled a relatively small number of racial/ethnic diverse and women veterans into the study. The general trend of racial/ethnic diversity in our sample, including representation of Asian identity in North California, Black and White identity in Arkansas, and White identity in Maine and Northern California, corresponds with the demographic characteristics of the veteran population in these geographic regions [59–61]. Additionally, we purposively sampled across VISNs and combined data across sites; therefore, our sample size is not sufficient to conduct meaningful cross-site comparisons limiting our understanding of potential geographic and racial/ethnic variation.

We oversampled women veterans and enrolled a fair number of women (*n* = 16) into the study. However, our analysis could have been qualitatively more nuanced and rich if more women had participated in the study and shared their experiences and perspectives. Historically, servicewomen and women veterans have been underrepresented in research examining VA or DoD healthcare use [62, 63]. Women veterans are the fasted growing segment of VA healthcare users [64]; yet, they represent a small population of VA patients and are often underrepresented and potentially excluded from analysis [65]. Use of a context-specific or gender-sensitive sampling approach may have increased women’s participation in our study [65]. Women veterans differ from men in healthcare utilization, how their VA care is organized (e.g., concentrated care in women only clinics), and gender-specific demands (e.g., primary caregivers), thus a one-size fits all approach to recruitment may limit women’s research participation. Future research should engage women’s health program leaders such as medical directors and program managers to identify recruitment strategies that are context-specific and therefore identify how and where women access healthcare and characterize their healthcare utilization patterns, as well as are sensitive to the unique demographics and healthcare experiences of women veterans. Such an approach would likely result in a gender-specific rather than gender-neutral recruitment approach and potentially two or more distinct recruitment strategies.

Finally, some veterans struggled to understand the purpose of the interpretation task and found it challenging to provide explanations for the clustering of items within the five dimensions of the shared cultural domain. After adjusting the instructions to include a brief hypothetical example, veterans more readily understood the task and offered important insight into how barriers within dimensions played out in their or other veterans’ healthcare experiences. Therefore, concrete instructions and an initial example are needed to successfully employ these methods.

## Conclusion

Our study findings reinforce the value of eliciting veteran’s perspectives on barriers to VA healthcare service use. By using participatory methods to elicit participants’ perspectives, we obtained veteran-centric barriers to care that are embedded in VA healthcare experiences and offer insight on how to decrease barriers to VA mental healthcare services use. The CDA approach outlined in this paper places patients at the forefront of data generation, analysis, and interpretation, which results in a shared understanding of barriers to care grounded in veterans’ experiences. Empirical, patient-centered data are critical to informing healthcare policy as well as developing valid measurement tools to accurately measure veterans’ experiences of barriers to care; these data can inform instrument domains and provide vocabulary, idiomatic expressions, and scenarios shared among patient populations [66].

## Additional file

Additional file 1: Free list activity interview guide. This interview guide elicits free list items within a cultural domain. The task takes about 10 min to complete. The guide includes a prompt that asks participants to list all the things they think of when they hear the prompt. The goal is to elicit words and brief phrases related to the prompt question, which then began items for further analysis. (DOCX 14 kb)

## Abbreviations

CBOC: Community-Based Outpatient Clinic
CDA: Cultural Domain Analysis
DoD: Department of Defense
MDS: Multidimensional Scaling
PTSD: Post-traumatic Stress Disorder
SOTA: State of the Art
VA: Veterans’ Administration
VISN: Veterans Integrated Service Network

## References

[1] McCarthy JF, Blow FC, Valenstein M, Fischer EP, Owen RR, Barry KL, Hudson TJ, Ignacio RV (2007). Veterans affairs health system and mental health treatment retention among patients with serious mental illness: evaluating accessibility and availability barriers. Health Serv Res.

[2] Panangala SV (2015). Veterans medical care: FY 2016 appropriations. Congressional Research Service.

[3] Garcia HA, Finley EP, Ketchum N, Jakupcak M, Dassori A, Reyes SC (2014). A survey of perceived barriers and attitudes toward mental health care among OEF/OIF veterans at VA outpatient mental health clinics. Mil Med.

[4] Wierwille JL, Pukay-Martin ND, Chard KM, Klump MC (2016). Effectiveness of PTSD telehealth treatment in a VA clinical sample. Psychol Serv.

[5] Desai MU, Pavlo AJ, Davidson L, Harpaz-Rotem I, Rosenheck R (2016). "I want to come home": Vietnam-era veterans' presenting for mental health care, roughly 40 years after Vietnam. Psychiatr Q.

[6] Sayer NA, Friedemann-Sanchez G, Spoont M, Murdoch M, Parker LE, Chiros C, Rosenheck R (2009). A qualitative study of determinants of PTSD treatment initiation in veterans. Psychiatry.

[7] Abraham T, Cheney AM, Curran GM (2015). A Bourdieusian analysis of U.S. military culture ground in the mental help-seeking literature. Am J Mens Health.

[8] Hoge CW, Castro CA, Messer SC, McGurk D, Cotting DI, Koffman RL (2008). Combat duty in Iraq and Afghanistan, mental health problems and barriers to care. US Army Med Dep J.

[9] Fischer EP, McSweeney JC, Wright P, Cheney A, Curran GM, Henderson K, Fortney JC (2016). Overcoming barriers to sustained engagement in mental health care: perspectives of rural veterans and providers. J Rural Health.

[10] Tai-Seale M, Sullivan G, Cheney A, Thomas K, Frosch D (2016). The language of engagement: "Aha!" moments from engaging patients and community partners in two pilot projects of the Patient-Centered Outcomes Research Institute. Perm J.

[11] Gerteis M (1993). Picker/commonwealth program for patient-centered care. Through the patient's eyes : understanding and promoting patient-centered care, 1st edn.

[12] Bardes CL (2012). Defining "patient-centered medicine". N Engl J Med.

[13] Smith RT, True G (2014). Warring identities: identity conflict and the mental distress of American veterans of the wars in Iraq and Afghanistan. Soc Ment Health.

[14] Putsch RW, Joyce M, Walker HK, Hall WD, Hurst JW (2011). Dealing with patients from other cultures. Clinical Methods: The History, Physical, and Laboratory Examinations.

[15] Tave TT, Wyers DR, Schreiber-Jones C, Fogger SA, McGuinness TM (2017). Improving quality outcomes in veteran-centric care. J Psychosoc Nurs Ment Health Serv.

[16] York J, Sternke LM, Myrick DH, Lauerer J, Hair C (2016). Development of veteran-centric competency domains for psychiatric-mental health nurse practitioner residents. J Psychosoc Nurs Ment Health Serv.

[17] Sestito SF, Rodriguez KL, Saba SK, Conley JW, Mitchell MA, Gordon AJ. Homeless Veterans' experiences with substance use, recovery, and treatment through photo elicitation. Subst Abus. 2017;38(4):422–31. 10.1080/08897077.2017.1356422. 10.1080/08897077.2017.1356422 28726549

[18] Bauer MS, Williford WO, McBride L, McBride K, Shea NM (2005). Perceived barriers to health care access in a treated population. Int J Psychiatry Med.

[19] Kilbourne AM, McCarthy JF, Post EP, Welsh D, Pincus HA, Bauer MS, Blow FC (2006). Access to and satisfaction with care comparing patients with and without serious mental illness. Int J Psychiatry Med.

[20] Hoge CW, Castro CA, Messer SC, McGurk D, Cotting DI, Koffman RL (2004). Combat duty in Iraq and Afghanistan, mental health problems, and barriers to care. N Engl J Med.

[21] Kim PY, Britt TW, Klocko RP, Riviere LA, Adler AB (2011). Stigma, negative attitudes about treatment, and utilization of mental health care among soldiers. Mil Psychol.

[22] Schensul JJ, LeCompte MD. Specialized ethnographic methods : a mixed methods approach. Lanham: AltaMira Press; 2013.

[23] Dressler WW, Oths KS, Bernard HR, Gravlee CC (2014). Social survey methods. Handbook of Methods in Cultural Anthropology.

[24] Bernard HR (2011). Research methods in anthropology : qualitative and quantitative approaches.

[25] Borgatti S, Schensul JJ, LeCompte MD, Alfred Hess G, Nastasi BK, Berg MJ, Williamson L (1998). Elicitation techniques for cultural domain analysis. The Ethnographer's Toolkit.

[26] Borgatti S (1994). Cultural domain analysis. J Quant Anthropol.

[27] Kroenke K, Spitzer RL (2002). The PHQ-9: a new depression diagnostic and severity measure. Psychiatr Ann.

[28] Kroenke K, Spitzer RL, Williams JB (2001). The PHQ-9: validity of a brief depression severity measure. J Gen Intern Med.

[29] Spitzer RL, Kroenke K, Williams JB (1999). Validation and utility of a self-report version of PRIME-MD: the PHQ primary care study. Primary care evaluation of mental disorders. Patient health questionnaire. JAMA.

[30] Keen SM, Kutter CJ, Niles BL, Krinsley KE (2008). Psychometric properties of PTSD checklist in sample of male veterans. J Rehabil Res Dev.

[31] Bradley KA, DeBenedetti AF, Volk RJ, Williams EC, Frank D, Kivlahan DR (2007). AUDIT-C as a brief screen for alcohol misuse in primary care. Alcohol Clin Exp Res.

[32] Patton MQ (2001). Qualitative research and evaluation methods.

[33] Treweek S, Shaun T, Elizabeth M, Marie P, Jonathan C, Monica K, Taina T, Marit J, Frank S, Sue W (2010). Strategies to improve recruitment to randomised controlled trials. Protocols.

[34] Junghans C, Feder G, Hemingway H, Timmis A, Jones M (2005). Recruiting patients to medical research: double blind randomised trial of "opt-in" versus "opt-out" strategies. BMJ.

[35] Hunt KJ, Shlomo N, Addington-Hall J (2013). Participant recruitment in sensitive surveys: a comparative trial of 'opt in' versus 'opt out' approaches. BMC Med Res Methodol.

[36] Palinkas LA, Horwitz SM, Green CA, Wisdom JP, Duan N, Hoagwood K (2015). Purposeful sampling for qualitative data collection and analysis in mixed method implementation research. Admin Pol Ment Health.

[37] Romney AK, Weller SC, Batchelder WH (1986). Culture as consensus: a theory of culture and informant accuracy. Am Anthropol.

[38] Weller SC (1987). Shared knowledge, intracultural variation, and knowledge aggregation. Am Behav Sci.

[39] Weller SC, Romney AK (1988). Systematic data collection.

[40] Burke JG, O'Campo P, Peak GL, Gielen AC, McDonnell KA, Trochim WMK (2005). An introduction to concept mapping as a participatory public health research method. Qual Health Res.

[41] Brewer DD (2002). Supplementary interviewing techniques to maximize output in free listing tasks. Field Methods.

[42] Dressler W, Borges C, Balieiro M, dos Santos J (2005). Measuring cultural consonance: examples with special reference to measurement theory in anthropology. Field Methods.

[43] Analytic Technologies: Anthropac. http://www.analytictech.com/anthropac/anthropac.htm. Accessed 19 July 2018.

[44] Davison ML. Multidimensional scaling. Santa Monica: Krieger Publishing Company; 1983.

[45] Kruskal JB (1964). Multidimensional-scaling by optimizing goodness of fit to a nonmetric hypothesis. Psychometrika.

[46] Rosas SR, Kane M (2012). Quality and rigor of the concept mapping methodology: a pooled study analysis. Eval Program Plann.

[47] Hout MC, Papesh MH, Goldinger SD (2013). Multidimensional scaling. Wiley Interdiscip Rev Cogn Sci.

[48] Verbi Software-Consult: MAXQDA software for qualitative data analysis, 1989–2016. In*.*; 2016.

[49] Ryan G, Bernard H (2003). Techniques to identify themes. Field Methods.

[50] Corbin JM, Strauss AL. Basics of qualitative research: techniques and procedures for developing grounded theory. Santa Monica: Sage Publications, Inc.; 2008.

[51] Kleinpell R, Buchman TG (2014). The value and future of patient-centered outcomes research. Critical Connections vol 2014.

[52] Fortney JC, Burgess JF, Bosworth HB, Booth BM, Kaboli PJ (2011). A re-conceptualization of access for 21st century healthcare. J Gen Intern Med.

[53] Koenig CJ, Maguen S, Monroy JD, Mayott L, Seal KH (2014). Facilitating culture-centered communication between health care providers and veterans transitioning from military deployment to civilian life. Patient Educ Couns.

[54] Tanielian T, Woldetsadik MA, Jaycox LH, Batka C, Moen S, Farmer C, Engel CC (2016). Barriers to engaging service members in mental health care within the U.S. military health system. Psychiatr Serv.

[55] Interagency Care Coordination Commitee (IC3). http://www.moaa.org/uploadedFiles/Content/Take_Action/Warrior_Family_Roundtable/IC3_One_Pager.pdf. Accessed 19 July 2018.

[56] Tanielian T, Hansen ML, Martin LT, Grimm G, Ogletree C. Supporting the mental health needs of veterans in the metro Detroit area. Santa Monica: RAND Corporation; 2016. 10.7249/rhq-06-01-15 PMC5158279 28083443

[57] Isaacs AR (1997). Vietnam shadows : the war, its ghosts, and its legacy.

[58] Finley EP (2011). Fields of combat : understanding PTSD among veterans of Iraq and Afghanistan.

[59] VETPOP2016: Living Veterans by State, Race/Ethnicity, Gender, 2015-2045. https://www.va.gov/vetdata/veteran_population.asp. Accessed 19 July 2018.

[60] QuickFacts: Mendocino County, California; Humboldt County, California. https://www.census.gov/quickfacts/fact/table/mendocinocountycalifornia,humboldtcountycalifornia/RHI125216#viewtop. Accessed 19 July 2018.

[61] California Veterans, By The Numbers. http://www.trbas.com/media/media/acrobat/2008-11/43592321.pdf. Accessed 19 July 2018.

[62] Bean-Mayberry B, Goldzweig C, Washington DL, Yano E, Batuman F, Huang C, Miake-Lye I, Shekelle P (2011). Updated systematic review of the literature on women Veteran's health. J Gen Intern Med.

[63] Goldzweig CL, Balekian TM, Rolon C, Yano EM, Shekelle PG (2006). The state of women veterans' health research. J Gen Intern Med.

[64] Yano EM, Bastian LA, Frayne SM, Howell AL, Lipson LR, McGlynn G, Schnurr PP, Seaver MR, Spungen AM, Fihn SD (2006). Toward a VA Women's Health Research agenda: setting evidence-based priorities to improve the health and health care of women veterans. J Gen Intern Med.

[65] Yano EM, Hayes P, Wright S, Schnurr PP, Lipson L, Bean-Mayberry B, Washington DL (2010). Integration of women veterans into VA quality improvement research efforts: what researchers need to know. J Gen Intern Med.

[66] Penn Handwerker W. Quick ethnography. Santa Monica: Rowman Altamira; 2001.

