# Supplementary material for: Veteran-centered barriers to VA mental healthcare services use
Source: BMC Health Serv Res. 2018 Jul 31;18:591. doi: 10.1186/s12913-018-3346-9 (PMC6069794; doi:10.1186/s12913-018-3346-9)
Supplement: Supplementary file 1 — Free list activity interview guide. This interview guide elicits free list items within a cultural domain. The task takes about 10 min to complete. The guide includes a prompt that asks participants to list all the things they think of when they hear the prompt. The goal is to elicit words and brief phrases related to the prompt question, which then began items for further analysis. (DOCX 14 kb) [file 12913_2018_3346_MOESM1_ESM.docx]

**Free list Activity**

**Introduction.**

I would like you to listen to the following prompt, take some time to think, and then tell me, using **words or brief phrases**, all the things that come to mind.

Do you have any questions before we begin?

**Prompt:** List all the things that you think can make it more difficult for veterans to get help for stress-related and emotional health problems.

(1) _________________________________

(2) _________________________________

(3) _________________________________

(4) _________________________________

(5) _________________________________

(6) _________________________________

(7) _________________________________

(8) _________________________________

(9) _________________________________

(10) _________________________________

[*Instructions to interviewer*: After the participant has listed items, use probes to elicit additional items.]

1.) Non-specific prompting:

- “What other kinds of things that might make it hard for veterans to get help?”

2.) Reading back free lists

- Slowly read back list, under the guise of checking for accuracy of written record.
- Then once again, prompt nonspecifically.
